# Neuropsychiatric symptom profile in neurocognitive disorders and their relationship with functional decline

**DOI:** 10.3389/fneur.2026.1805908

**Published:** 2026-07-06

**Authors:** Carolyn W. Zhu, Lon S. Schneider, Gregory A. Elder, Laili Soleimani, Judith Neugroschl, Corbett Schimming, Mary Sano

**Affiliations:** 1 Brookdale Department of Geriatrics and Palliative Medicine, Icahn School of Medicine at Mount Sinai, New York, NY, United States; 2 James J Peters VA Medical Center, Bronx, NY, United States; 3 Alzheimer Disease Research Center, Department of Psychiatry, Icahn School of Medicine at Mount Sinai, New York, NY, United States; 4 Department of Psychiatry, Neurology, and Gerontology, Keck School of Medicine and Leonard Davis School of Gerontology, University of Southern California, Los Angeles, CA, United States

**Keywords:** Alzheimer’s disease, frontal temporal lobe dementia, Lewy body disease, neurocognitive disorders, neuropsychiatric symptoms, functional decline

## Abstract

**Introduction:**

Neuropsychiatric symptoms (NPS) are common in neurocognitive disorders (NCD) and are known to negatively impact patient’s functional abilities. However, our understanding of the relationship between individual NPS and functional decline in patients with NCD across the spectrum of cognitive impairment is limited. Here we examine the relationship between specific NPS to characterize their effects on patient’s function within and across different etiologies.

**Method:**

Longitudinal observational study using the National Alzheimer’s Coordinating Center Uniform Data Set (NACCUDS). We examined NPS as characterized by expert clinicians and report its impact on the outcome of functional decline, measured by the Functional Assessment Questionnaire (FAQ), a standardized assessment of activities of daily living, by dementia etiology (Alzheimer’s disease (AD, *N* = 11,044), Lewy Body Disease (LBD, *N* = 921), and behavioral variant frontal temporal lobe dementia (bvFTD, *N* = 933)).

**Results:**

We find apathy was the most commonly endorsed and the most persistent symptom across dementia types in all groups and was associated with more rapid functional decline in AD and bvFTD. On the contrary, depression, occurring in 40% or more of all groups, was not associated with worsening functional impairment in any group. We identified patterns that indicated higher rates of disinhibition and persistent disinhibition in bvFTD compared to AD and LBD. Psychosis had unique impact on functional decline in AD and LBD as did agitation in AD.

**Discussion:**

Differential impact of individual NPS across dementia etiologies and their impact on functional decline may have important consequences for clinical trial designs for the treatment of these symptoms.

## Introduction

Neuropsychiatric symptoms (NPS), including apathy, depression, anxiety, irritability, agitation, aggression, delusions, hallucinations, and sleep and appetite disturbances are common across neurocognitive disorders (NCDs). They are associated with worse patient outcomes and have profound consequences on patients, families and caregivers, and society (1–8). Most patients develop at least some NPS during the disease course, however prevalence and persistence of individual symptoms are highly variable (9–16). Symptoms wax and wane and may present intermittently, disappear, and recur throughout the illness course (13, 17, 18).

Prevalence of individual symptoms varies across NCD. For example, apathy, one of the most common NPS in dementia (13, 16, 19, 20), is experienced by 24 to 85% of patients with AD dementia, 35 to 100% with LBD, and 50 to 100% with FTD (21). Psychosis, including hallucinations and delusions, are more likely to be experienced in patients with LBD (22). Disinhibition is most common in those with bvFTD (42.3%) compared with AD (30.7%) and LBD (26.3%) (23).

Our understanding of the relationship between individual NPS and functional decline in patients with NCD across the spectrum of cognitive impairment is limited. In an earlier study, we examined the relationships between individual symptoms and functional decline in AD (24). Results showed that apathy was strongly related to accelerated functional decline in AD, and to a somewhat lesser extent, agitation and delusions were as well. Several earlier studies also showed relationship between apathy and faster rate of functional decline in LBD and bvFTD as well (25, 26). However, other NPS were not included in those studies. A recent study examining the relationships between individual NPS and functional impairment in early-stage bvFTD found specific NPS, particularly apathy and disinhibition, predicted subsequent functional decline (27). In the current longitudinal observational study, we extend this line of inquiry and examine relative contribution of individual NPS on functional decline across AD, LBD, and bvFTD. Consistent data sources and methodologies used in these studies allow comparison of individual symptoms in AD, bvFTD and LBD. Understanding the course of individual NPS and identifying their relationships with functional decline within each NCD is vitally important for planning targeted interventions for better disease management and treatment.

## Methods

### Data source and sample derivation

Data are from the National Alzheimer’s Coordinating Center Uniform Data Set (NACCUDS). Recruitment, participant evaluation, and diagnostic criteria have been detailed elsewhere (28). Beginning in September 2005, the start date for the NACCUDS, participants have been enrolled and followed prospectively at approximately 12-month intervals from National Institute of Aging funded Alzheimer’s Disease Centers (ADCs) located throughout the United States with a standardized protocol.

At each visit, participants receive a clinical diagnoses made by expert clinicians based on up-to-date research diagnostic criteria, using standardized neuropsychiatric battery and all available information including patient exam, informant-provided history, cognitive testing, neuroimaging, and genetic profile according to NACCUDS procedures^1^ (29).

**Figure 1:**
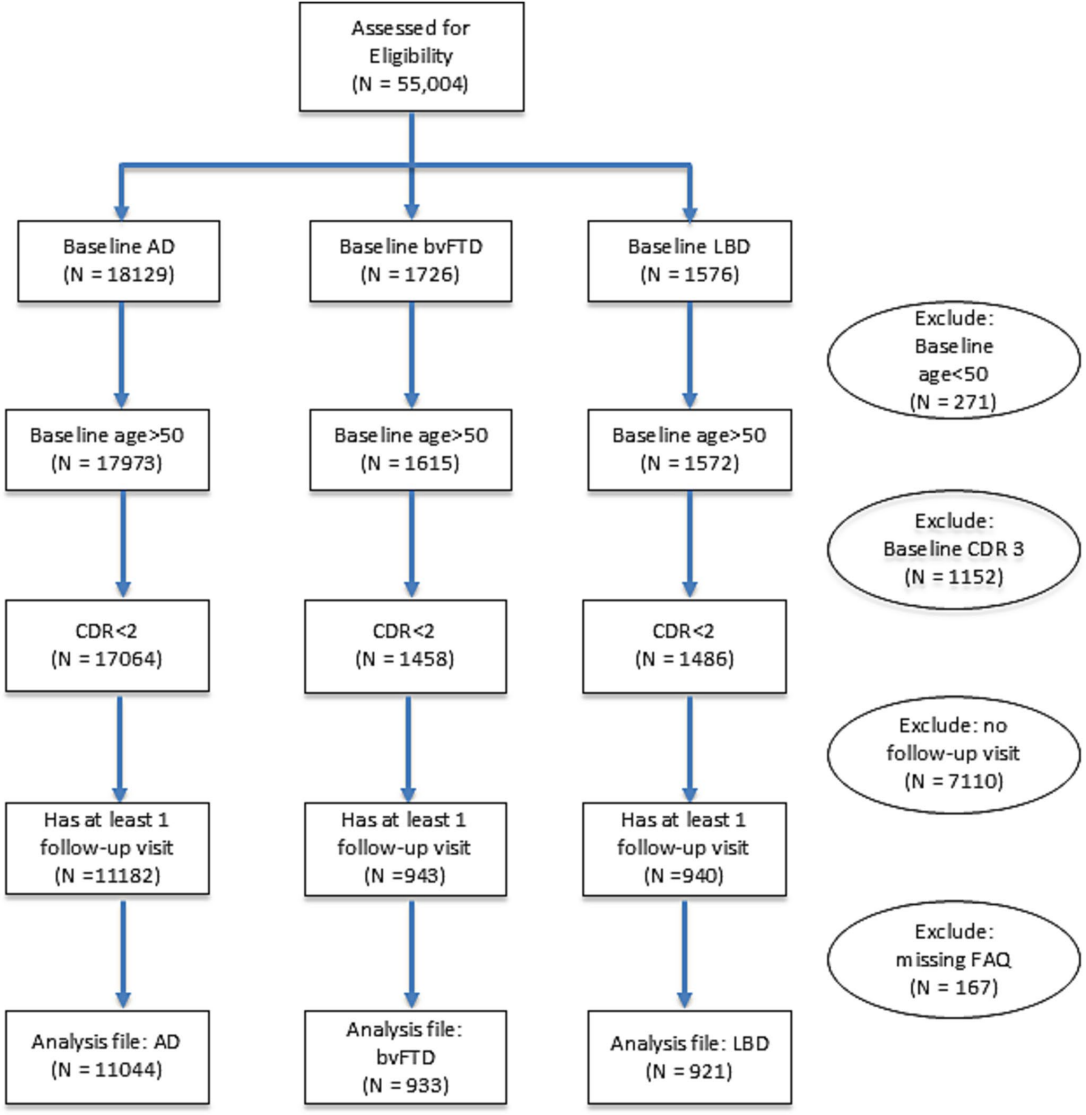
Sample selection flow chart.

We used the same sample identification algorithms in our earlier studies to define the three NCD cohorts. Specifically, participants with AD included those with a primary etiologic diagnosis of AD at baseline (24, 30). Participants with LBD included those with a primary diagnosis of LBD at baseline following the McKeith criteria as described in the NACCUDS Guidebook (31) and described in detail in an earlier study (25). Participants with bvFTD included those who had a primary clinical diagnosis of FTLD with behavioral variant FTD (bvFTD) at baseline (etiology = FTLD, NACCBVFT = behavioral variant FTD) (26). To be included in the current study, participants had to be (1) age 50 or older at NACCUDS enrollment between September 2005 and September 2025 data freeze, (2) cognitive status of mild cognitive impairment (MCI) or dementia, and (3) had at least one follow-up visit. Sample derivation is presented in Figure 1.

Written informed consent was provided by all participants and informants and approved by local Institutional Review Boards (IRB). Research using the NACCUDS database was approved by the University of Washington IRB. This study followed the STROBE (Strengthening the Reporting of Observational Studies in Epidemiology) reporting guideline.

### Data sharing

The data that support the findings of this study are openly available at https://naccdata.org/data-collection/forms-documentation/uds-3.

### Outcome: function

Our sole dependent variable in the current study is participants’ function, measured using the Functional Assessment Questionnaire (FAQ) reported from interviews with study partners (32). The FAQ asks whether the participant had any difficulty or needed help with 10 items in the previous 4 weeks on a scale from 0 to 3, corresponding to normal (0), has difficulty but does by oneself (1), requires assistance (2), and dependent (3). Responses to each item were summed to obtain a total FAQ score (range = 0–30). About 1% of participants (*N* = 167, 138 AD, 10 bvFTD, 19 LBD) were reported to have not attempted any tasks and therefore had a missing value for all FAQ items were excluded from the analysis.

### Neuropsychiatric symptom (NPS) profiles

At each visit, study clinicians provided a clinical assessment of whether a participant currently manifested the symptom as a meaningful change in behavior (yes = 1, no = 0) from a list of NPS using the NACCUDS B9 Form. The following symptoms were assessed: apathy-withdrawal, depression, visual/auditory hallucinations, abnormal/false/delusional beliefs, disinhibition, irritability, and agitation. All NPS symptoms were asked consistently over time following NACCUDS protocol. The NACC Guidebook instructs the clinicians to provide a clinical determination of symptoms based on all available information obtained through participant, informant, medical records and/or observations^2^ (31, 33). We constructed a psychosis indicator by combining visual/auditory hallucinations and abnormal/false/delusional beliefs following International Psychogeriatric Association (IPA) (34).

Based on how often a participant was reported by clinician judgment to have a symptom throughout the follow-up period, we categorized the symptom into mutually exclusive groups: (1) never occurred across all visits, (2) single episode, (3) intermittently occurring (more than one but <50% visits), (4) persistently occurring (≥50% visits), and (5) always occurred across all visits. We had previously reported that worse NPS profile groups were strongly correlated with worse baseline function and also with faster rate of functional decline in AD (24). However, aside from those whose NPS was never reported at any visit, models did not reveal statistically significant differences in the trajectories of functional decline by NPS profiles in bvFTD and LBD. We therefore used an indicator for any occurrence of each symptom during the study in the estimation models.

### Dementia severity

Participants’ baseline dementia severity was assessed using Clinical Dementia Rating (CDR) Scale (35). The global CDR (range = 0–3) is a widely used dementia staging scale.

### Demographic and clinical characteristics

Demographic characteristics included age, sex, race/ethnicity, years of education, marital status, and living arrangement (living alone = yes/no). Participant medical history was obtained by clinician interview and review of medical records as reported to NACCUDS. Depressive symptoms were measured using the 15-item Geriatric Depression Scale (GDS-15) (36, 37). Medication use was reported using a medication inventory (Form A4) which included all medications (including nonprescription drugs, vitamins, and supplements) taken by the participant within 2 weeks of their visit. A total number of medications taken was reported in the NACCUDS and was used in the analyses as a proxy for medications burden and comorbidities.

### Statistical analyses

We first reported baseline demographic and clinical characteristics and NPS profiles by the three diagnosis groups.

We then estimated the relationship between each NPS and functional decline over time using linear mixed models (LMM) which allows for inclusion of participants with incomplete follow-up. The main independent variables were indicators for any occurrence of each NPS during the study and their interactions with time (years since baseline), calculated as the difference between visit date and baseline date and expressed in years. All models controlled for baseline dementia severity, time, and their interactions. A quadratic term on time was included to assess potential non-linearity in change in function over time. Models also included the following covariates: baseline age, gender, race/ethnicity, years of education, referral source (professionals vs. other), total number of visits, indicators for history of diabetes, hypertension, Apolipoprotein (ApoE) ε4 allele (none, heterozygote, homozygote), and total number of medications used as reported in the NACCUDS.

All models included subjects and ADCs as random intercepts, assuming participants were nested within each ADC. We tested models that included individual random slopes to allow participants to differ in their overall rate of change over time. However, estimated coefficients of the fixed effects were substantively different from the more complex random-slopes models. Likelihood ratio tests suggested that including a random slope did not significantly improve model fit. Because of these reasons, random slopes were dropped and the more parsimonious random-intercept models were used. Initial models also included interaction terms between CDR and NPS. None of the interaction terms were statistically significant and were subsequently dropped. We examined the stability of results between NPS profiles and function in each group using subsamples of participants who had completed at least 3 or 4 visits to mitigate the issue of short follow-up. Results were substantively similar and presented in Supplementary Tables and Figures. Models were estimated for each diagnostic group separately. All analyses were performed using Stata 19.0 (38). Statistical significance was set *a priori* at *p* < 0.05.

## Results

### Baseline characteristics

The sample included 11,044 participants with AD, 933 with bvFTD, and 921 with LBD (Table 1). Mean ± SD age of participants with AD was 73.5 ± 9.1 years, with 15.0 ± 3.5 years of education. Participants were 47.7% male and largely White (83.1%) and non-Hispanic (91.8%). 57.4% had CDR 0.5, 34.0% CDR 1, and 8.6% CDR 2. Participants were moderately impaired cognitively, with low depressive symptoms: mean ± SD MMSE of 23.1 ± 5.2, FAQ 10.7 ± 8.6, GDS 2.4 ± 2.5. Participants took an average of 6.4 medications. Cardiovascular conditions were common with 53.5% having hypertension and 12.7% having diabetes. Average follow-up was 4.1 ± 2.4 years. 56.8% carried at least one ApoE ε4 allele.

**Table 1:** Baseline characteristics by diagnostic group.

Variables	AD	bvFTD	LBD
*N*	11,044	933	921
Age, mean (SD)	73.5 (9.1)	64.7 (7.9)	71.5 (7.6)
Male, (%)	47.7	63.1	79.3
Race, (%)			
White	83.1	94.6	92.5
Black/African American	11.4	1.5	4.7
All other	5.5	3.9	2.8
Hispanic ethnicity, (%)	8.2	3.5	4.3
Years of education, mean (SD)	15.0 (3.5)	15.5 (3.1)	15.6 (3.3)
Years of follow up, mean (SD)	4.1 (2.4)	3.8 (2.3)	3.6 (1.8)
CDR global, (%)			
0.5	57.4	46.2	63.7
1	34.0	40.3	28.4
2	8.6	13.5	7.8
FAQ, mean (SD)	10.7 (8.6)	13.3 (8.7)	11.0 (8.5)
GDS, mean (SD)	2.4 (2.5)	3.2 (3.1)	3.9 (3.1)
MMSE, mean (SD)	23.1 (5.2)	23.7 (5.8)	25.0 (4.7)
Number of ApoE ε4 alleles, (%)			
0	43.1	69.6	60.1
1	43.7	26.8	35.0
2	13.1	3.6	4.9
Diabetes, (%)	12.7	11.7	11.3
Hypertension, (%)	53.5	47.6	49.0
Professional referral source (%)	59.7	76.3	74.6
Total number of medications, mean (SD)	6.4 (4.0)	5.7 (3.9)	7.6 (4.6)

AD, Alzheimer’s disease; bvFTD, behavioral variant frontal temporal lobe dementia; LBD, Lewy Body Disease; CDR, Clinical Dementia Rating; FAQ, Functional Assessment Questionnaire; GDS, Geriatric Depression Scale; MMSE, Mini-mental State Examination. ApoE, Apolipoprotein.

Mean ± SD age of participants with bvFTD was 64.7 ± 7.9 years, with 15.5 ± 3.1 years of education. The cohort was largely male (63.1%) and overwhelmingly White (94.6%) and non-Hispanic (96.5%). 46.2% at CDR 0.5 40.3% at CDR 1 13.5% at CDR 2. Clinical measures suggest moderate cognitive and functional impairment and low depressive symptoms, with mean ± SD MMSE 23.7 ± 5.8, FAQ 13.3 ± 8.7, GDS 3.2 ± 3.1. Participants took an average of 5.7 ± 3.9 medications. Cardiovascular conditions were common with 47.6% having hypertension and 11.7% having diabetes. Average follow-up was 3.8 ± 2.3 years. Majority of the participants (69.6%) do not carry any ApoE ε4 alleles.

Mean ± SD age of participants with LBD was 71.5 ± 7.6 years, with 15.6 ± 3.3 years of education. The cohort was predominantly male (79.3%) and overwhelmingly White (92.5%) and non-Hispanic (95.7%). 63.7% had CDR 0.5, 28.4% had CDR 1 and 7.8% had CDR 2. Clinical measures were slightly better than AD and bvFTD groups, with mean ± SD MMSE 25.0 ± 4.7 and FAQ 11.0 ± 8.5. GDS was slightly higher at 3.9 ± 3.1. Participants took an average of 7.6 ± 4.6 medications. Cardiovascular conditions were common with 49.0% having hypertension and 11.3% having diabetes. Average follow-up was 3.6 ± 1.8 years. 40% carried at least one ApoE ε4 allele.

### NPS profile by clinician judgment

**Figure 2:**
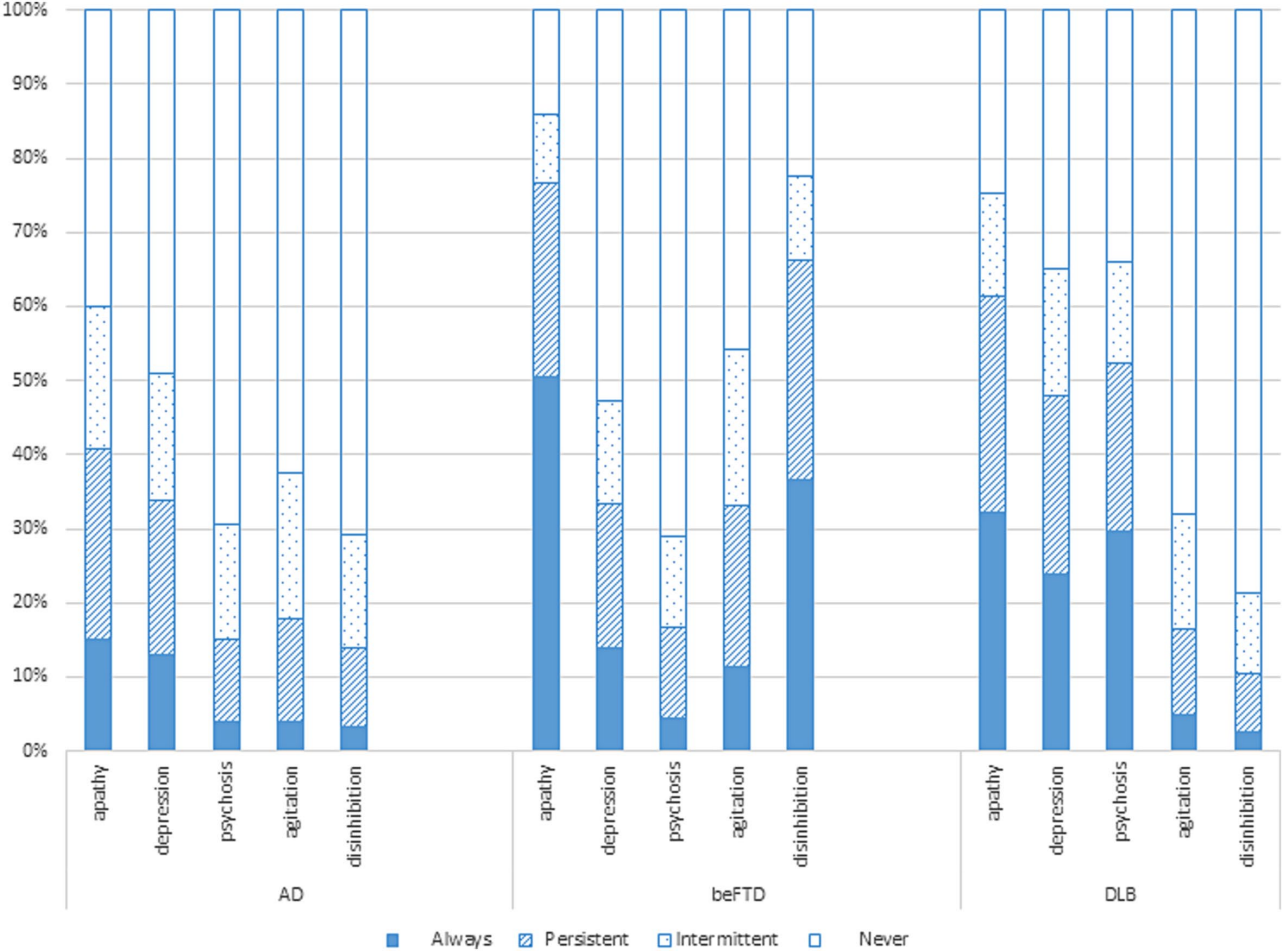
NPS profile by diagnostic group. NPS grouped into mutually exclusive groups: never occurred across all visits, intermittently occurring (more than one but <50% visits), persistently occurring (≥50% visits), and always occurred across all visits.

Profiles of individual NPS by diagnostic group are shown in Figure 2. In participants with AD, apathy was the most commonly endorsed symptom throughout the follow-up period (60%), followed by depressed mood (51%). Apathy also was the most persistently endorsed NPS throughout the follow-up period with 40.7% of participants endorsed by the clinician as having apathy more than half of the visits. In participants with bvFTD, apathy also was the most commonly endorsed symptom throughout the follow-up period (86%), followed by disinhibition (77.6%) and agitation (54.2%). Apathy and disinhibition also were the most persistently endorsed NPS, with half of the participants always having apathy (50.4%) and more than a third (36.6%) always having disinhibition. In participants with LBD, apathy remained the most commonly endorsed symptom throughout the follow-up period (75.4%), followed by psychosis (66.2%) and depressed mood (65.2%). Apathy, psychosis, and depressed mood also were the most persistent NPS.

### Estimated relationships between NPS, baseline CDR, and function

**Figure 3:**
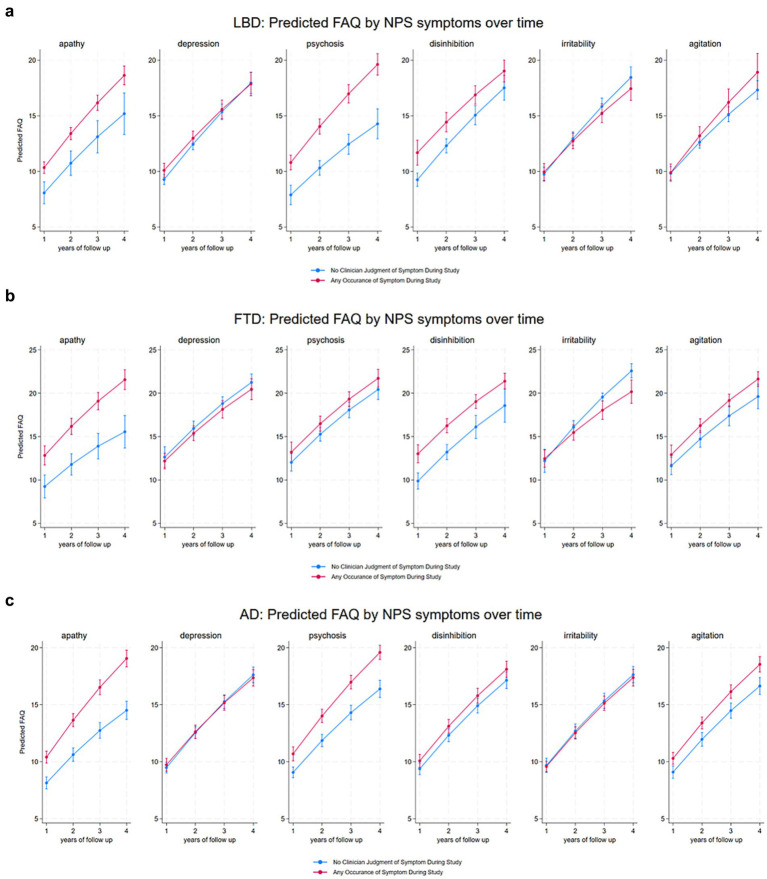
**(a)** Predicted FAQ by NPS profile over time in AD. **(b)** Predicted FAQ by NPS profile over time in bvFTD. **(c)** Predicted FAQ by NPS profile over time in LBD.

Adjusted LMM estimation results on the relationships between function and each NPS over time for each diagnostic group are shown in Table 2. Predicted FAQ scores generated from these model estimates are presented in Figure 3.

**Table 2:** Multivariable results from Linear mixed models (LMM) of the independent relationship between each NPS and functional decline over time in AD, bvFTD, and LBD.

Variables	AD			bvFTD			LBD		
	Coefficient	SE	*p*	Coefficient	SE	*p*	Coefficient	SE	*p*
Years of follow-up (time)	3.104	(0.143)	≤0.001	4.090	(0.627)	≤0.001	3.109	(0.374)	≤0.001
Years of follow-up squared	−0.178	(0.009)	≤0.001	−0.219	(0.026)	≤0.001	−0.149	(0.034)	≤0.001
Baseline dementia severity (ref: CDR = 0.5)									
CDR = 1	9.804	(0.401)	≤0.001	7.007	(0.692)	≤0.001	7.489	(1.342)	≤0.001
CDR = 2	17.358	(0.497)	≤0.001	14.402	(1.188)	≤0.001	15.837	(1.515)	≤0.001
Interaction between baseline CDR * Time									
CDR = 1	−0.498	(0.088)	≤0.001	−0.261	(0.149)	0.080	−0.349	(0.311)	0.261
CDR = 2	−1.713	(0.116)	≤0.001	−1.372	(0.330)	≤0.001	−1.806	(0.322)	≤0.001
Effects on baseline FAQ from clinician judged NPS vs. never having the symptom									
Apathy	1.502	(0.206)	≤0.001	2.772	(0.910)	0.002	1.888	(0.535)	≤0.001
Depression	0.415	(0.213)	0.051	−0.345	(0.471)	0.464	1.132	(0.436)	0.009
Psychosis	1.097	(0.251)	≤0.001	1.138	(0.653)	0.081	2.099	(0.804)	0.009
Disinhibition	0.610	(0.245)	0.013	3.252	(0.556)	≤0.001	2.742	(0.891)	0.002
Irritability	−0.049	(0.244)	0.841	1.135	(0.890)	0.202	0.600	(0.637)	0.347
Agitation	0.970	(0.252)	≤0.001	1.045	(0.537)	0.052	−0.448	(0.665)	0.500
Effects on rate of change in FAQ from clinician judged NPS vs. never having the symptom									
Apathy	0.761	(0.097)	≤0.001	0.809	(0.287)	0.005	0.389	(0.236)	0.099
Depression	−0.169	(0.460)	0.522	−0.110	(0.181)	0.542	−0.305	(0.190)	0.109
Psychosis	0.527	(0.087)	≤0.001	0.038	(0.278)	0.890	0.810	(0.303)	0.007
Disinhibition	0.089	(0.064)	0.166	−0.108	(0.304)	0.722	−0.307	(0.307)	0.317
Irritability	−0.053	(0.079)	0.503	−0.885	(0.387)	0.220	−0.403	(0.189)	0.303
Agitation	0.232	(0.074)	0.002	0.244	(0.185)	0.186	0.509	(0.321)	0.112
Control variables									
Baseline age	0.042	(0.013)	0.002	0.068	(0.026)	0.010	0.086	(0.028)	0.002
Male	−1.374	(0.137)	≤0.001	−0.983	(0.377)	0.009	−0.759	(0.385)	0.049
Race/ethnicity (REFEREnce = Non-hispanic white)	1.546	(0.258)	≤0.001	0.668	(0.887)	0.452	−0.399	(0.635)	0.530
Years of education	−0.031	(0.021)	0.130	−0.005	(0.066)	0.937	0.067	(0.074)	0.362
NACCUDS version	−0.571	(0.222)	0.010	−0.486	(0.420)	0.247	−1.060	(0.773)	0.170
Years of follow up	−0.877	(0.063)	≤0.001	−0.778	(0.123)	≤0.001	−1.106	(0.099)	≤0.001
Lives alone at baseline	−1.428	(0.173)	≤0.001	−1.621	(0.841)	0.054	−2.005	(0.805)	0.013
Referred by professionals	0.696	(0.229)	0.002	0.136	(0.535)	0.799	−0.507	(0.388)	0.192
Diabetes	−0.402	(0.112)	≤0.001	−0.407	(0.605)	0.501	−1.641	(0.490)	0.001
Hypertension	−0.018	(0.130)	0.887	−0.137	(0.454)	0.763	−0.163	(0.467)	0.728
Number of medications	−0.017	(0.018)	0.351	−0.157	(0.049)	0.001	0.022	(0.034)	0.515
Apolipoprotein ε4 allele (ApoE ε4) (reference = No ε4)									
One ε4	0.652	(0.114)	≤0.001	−0.319	(0.283)	0.259	0.909	(0.383)	0.018
Two ε4s	1.317	(0.222)	≤0.001	2.765	(0.985)	0.005	0.623	(0.764)	0.415
ApoE missing	0.407	(0.277)	0.141	0.170	(1.025)	0.868	0.289	(0.633)	0.647

AD, Alzheimer’s disease; bvFTD, behavioral variant frontal temporal lobe dementia; LBD, Lewy Body Disease.

### Time trends and relationship with dementia severity

For all diagnostic groups, function declined over time but the rate of decline seemed to slow slightly. Estimated coefficients for CDR and its interaction with time showed that baseline function and rate of functional decline was worse for more severe dementia, and rate of functional decline was slower in those with severe dementia compared to mild dementia, possibly due to floor effects on poor function in those with severe dementia.

Specifically, in AD, FAQ scores worsened by an estimated average ± SE of 3.104 ± 0.143 points per year (*p* ≤ 0.001) with a slowing rate of decline by 0.178 ± 0.009 points per year (*p* ≤ 0.001). At baseline, compared with those with baseline CDR = 0.5, FAQ scores were 9.804 ± 0.401 (*p* ≤ 0.001) points higher (worse) in those with CDR = 1 and 17.358 ± 0.497 (*p* ≤ 0.001) points higher in those with CDR = 2. Rate of decline in function was slower by an estimated average of 0.498 ± 0.088 points per year in those with baseline CDR = 1 (*p* ≤ 0.001) and 1.713 ± 0.116 points per year in those with baseline CDR = 2 (*p* ≤ 0.001) compared to participants with baseline CDR = 0.5.

In bvFTD, FAQ scores worsened by an estimated average of 4.090 ± 0.627 points per year (*p* ≤ 0.001) with a slowing rate of decline by 0.219 ± 0.026 points per year (*p* ≤ 0.001). At baseline, compared with those with baseline CDR = 0.5, FAQ scores were 7.007 ± 0.692 (*p* ≤ 0.001) points higher (worse) in those with CDR = 1 and 14.402 ± 1.188 (*p* ≤ 0.001) points higher in those with CDR = 2. Rate of decline in function was slower by an estimated average of 1.372 ± 0.330 points per year in those with baseline CDR = 2 (*p* ≤ 0.001) compared to participants with baseline CDR = 0.5, suggesting a possible plateauing effect.

In LBD, FAQ scores worsened by an estimated average ± SE of 3.109 ± 0.374 points per year (*p* ≤ 0.001) with a slowing rate of decline by 0.149 ± 0.034 points per year (*p* ≤ 0.001). At baseline, compared with those with baseline CDR = 0.5, FAQ scores were 7.489 ± 1.342 (*p* ≤ 0.001) points higher (worse) in those with CDR = 1 and 15.837 ± 1.515 (*p* ≤ 0.001) points higher in those with CDR = 2. Rate of decline in function was slower by an estimated average of 1.806 ± 0.322 points per year in those with baseline CDR = 2 (*p* ≤ 0.001) compared to participants with baseline CDR = 0.5.

### Relationships between NPS and baseline function

In AD, results showed that after controlling for other NPS, compared to those who never had the symptom, baseline FAQ scores were higher in those who had apathy compared to those who never had apathy (mean ± SE = 1.502 ± 0.206, *p* ≤ 0.001), psychosis (1.097 ± 0.251, *p* ≤ 0.001), agitation (0.970 ± 0.252, *p* ≤ 0.001), and disinhibition (0.610 ± 0.245, *p* = 0.013), respectively. In bvFTD, results showed that compared to those who never had the symptom, baseline FAQ scores were higher in those who had apathy (2.772 ± 0.910, *p* = 0.002), and disinhibition (3.252 ± 0.556, *p* ≤ 0.001), respectively. In LBD, results showed that compared to those who never had the symptom, baseline FAQ scores were higher in those who had apathy compared to those who never had apathy (1.888 ± 0.535, *p* ≤ 0.001), depression (1.132 ± 0.436, *p* = 0.009), disinhibition (2.742 ± 0.891, *p* = 0.002), psychosis (2.099 ± 0.804, *p* = 0.009), respectively.

### Relationships between NPS and rate of functional decline over time

The interaction terms between each NPS and time estimated differences in the rate of change in FAQ over time in those who had the symptom at some point during the study compared to those who never had the symptom. In AD, results showed that rate of decline in function was faster in those with apathy (mean ± SE = 0.761 ± 0.097, *p* ≤ 0.001), psychosis (0.527 ± 0.087, *p* ≤ 0.001), and agitation (0.232 ± 0.074, *p* = 0.002) compared to those without the symptom. In bvFTD, rate of decline in function was faster in those with apathy (0.809 ± 0.287, *p* = 0.005) compared to those without the symptom. In LBD, rate of decline in function was faster in those with psychosis (0.810 ± 0.303, *p* = 0.007) compared to those without the symptom.

## Discussion

In this study, we examined the association between NPS with functional decline over time in a large cohort of extensively characterized research participants with AD, bvFTD, and LBD. Consistent with earlier studies, we found that apathy was the most common symptom in all groups and was associated with accelerated decline in function in AD and bvFTD, even after controlling for other symptoms. On the contrary, depression, occurring in 40% or more of all groups, was not associated with worsening functional impairment in any group. These results are consistent with earlier studies that showed apathy as an independent construct in AD distinct from depression in prevalence and in impact on patient outcomes (39, 40). In individuals with subjective cognitive decline, apathy and depression have been shown to have different trajectories over time, and apathy alone is more likely to be associated with conversion to dementia (41). Together these results highlight the importance of assessing for apathy at all stages of cognitive decline. We identified patterns that indicated higher NPS in bvFTD compared to AD and LBD with distinct patterns of specific symptoms in each diagnostic group. Distinct patterns in bvFTD included high rates of disinhibition with persistent disinhibition observed in nearly 60% in the group, much higher than either AD or LBD. The predominant pattern of disinhibition noted in this cohort was typical of this type of dementia and in this group was associated with baseline functional problems, but not with rate of functional decline. A different pattern, observed in the LBD group, showed the greatest persistence of psychotic features, and unlike in other dementias, these were associated with accelerated decline in function. Perhaps the psychotic features in LBD have a specific and distinctive neurobiology, leading to greater impact on functional decline. Understanding specific and independent effects of each NPS within each dementia etiology is a critical step to designing clinical trials to develop interventions for these disturbing symptoms.

As biomarkers become available to identify dementia etiology, precision treatment of specific dementias is becoming possible. Amyloid targeting therapies for AD have become available with the advent of amyloid biomarkers to identify appropriate candidates for treatment. However, there is little data on the impact of these treatments on NPS. A recent meta-analysis suggested lower depression with monocloncal antibodies against amyloid based on reported side effects, but individual studies have not always systematically assessed the impact of these drugs on NPS (42). This omission may be based on the mistaken impression that NPS are not common in MCI or mild dementia and structured measurement may not be part of pivotal trials. Biomarkers for other dementia etiologies are not yet available, but development is ongoing and could advance therapeutics for specific conditions. It is unknown if the biology of specific NPS is common across dementia etiologies. Such a possibility would permit targeted treatment for the underlying biology of the symptom across NCD. This could allow identifying a group for treatment regardless of underlying dementia etiology.

Some perspectives have assumed that common phenomenology defined by diagnostic criteria could be sufficient in the absence of a biomarker to guide pharmacological approaches to treatment independent of dementia etiology. The epidemiology and the association with clinical outcome of function found in this study suggest that this may be true for apathy or depression. However, the distinctively different patterns of symptoms such as disinhibition and psychosis in dementias of different etiologies suggest that the underlying biology may differ between dementia subtypes. If so, the same symptoms may need to be optimally treated in distinct diseases differently. Clinical trials also may need to be conducted within specific etiological groups. The present data suggest that effect sizes for clinical outcomes such as function could be distinctly different in by NCD etiology and adjustment in randomization for dementia etiology might be quite important.

While treatments for NPS in NCDs have demonstrated variable benefits, some interventions that have shown modest effects and are widely employed in clinical practice. Pharmacologic treatments, although associated with side effects, cognitive decline, and even mortality, are often required when non-pharmacologic interventions are ineffective or unavailable or when symptoms are severe, resistant, or present a danger to the patient or caregiver. An earlier study using the NACCUDS data showed no improvement in NPS associated with using atypical antipsychotics (43). Shorter term (6–12 week) clinical trials often do not include functional outcomes. A 26-wk trial with olanzapine of patients similar to patients in our cohort showed worsening cognition and behavior, suggesting that function may also have worsened. Our findings in this study raise the possibility that functional decline trajectories may be an important outcome measure.

An observation from this study is that the impact on function of a given symptom can be captured even in the presence of multiple symptoms. In fact, the presence of multiple symptoms is common. This can provide confidence in recruitment of those with comorbid NPS symptoms. Of note, pharmacologic interventions may have different, even opposing, effects on individual symptoms, complicating clinical trials designs. However, trials that include the widest range of participants (such as those with multiple NPS) will yield the most informative results for real world use. Further work might confirm these NPS patterns in populations of biologically defined dementia participants.

The study has a number of limitations. First, it is worth remembering that the NACCUDS data from which our analysis sample is derived is not representative of the general population. It is well-known that the NACCUDS is substantially less ethnic-racially diverse than the general population, with higher education levels, and has relatively fewer medical and psychiatric comorbidities. Specific to the current study, some of the tasks used in the FAQ to assess function may reflect individual’s educational and socioeconomic exposure. Individuals with higher education may have greater familiarity with and engagement in these activities, potentially leading to higher baseline performance and different rates of decline compared to more diverse populations. As a result, the trajectories identified in the current study may not generalize to individuals with lower educational attainment or differing socioeconomic backgrounds. It is also worth remembering that while the FAQ is widely accepted as a reliable and valid measure of function, it is based on informant-report and may have potential biases from informant’s perspectives. Second, as the current analysis is focused on function, we have not examined the relationship between behavioral symptoms and other outcomes. Potential benefits from treatment of behavioral symptoms on other outcomes are not discussed here. We operationalized a proxy measure of cumulative burden of NPS by assessing the persistence of NPS. While this approach does not follow strict temporal orders between exposure to NPS and functional outcome at each visit, because NPS are often fluctuating and episodic, looking at NPS profile across visits as constructed in the current analysis may improve the likelihood of capturing NPS. The binary measure that was used in the multivariable analysis may reduce misclassification and enhance statistical stability by assessing the persistence of symptoms. Results should not be interpreted as reflecting causal effects. Lastly, as the focus of the analysis is to explore patterns of relationships between NPS and function rather than examining any individual predetermined hypothesis, we did not perform statistical corrections for multiple comparisons. Well-known strengths of the studies using the NACCUDS include its large sample size and the long duration, extensive clinical characterization of participants including neuropsychological testing, functional assessment, and case ascertainment of behavioral symptoms by ADRC-based dementia expert clinicians utilizing informant input.

In conclusion, differential relationships of individual NPS across several neurocognitive disorders shown in this study boosts our confidence in the relationships between NPS, particularly apathy, and functional decline in neurocognitive disorders. These relationships between NPS and functional decline may have important consequences for clinical trial designs for the treatment of these symptoms.

## Data Availability

The datasets presented in this study can be found in online repositories. The names of the repository/repositories and accession number(s) can be found below: https://www.naccdata.org/.

## Footnotes

1
https://www.alz.washington.edu

2
https://naccdata.org/data-collection/training
