# Supplementary material for: Neuropsychiatric symptom profile in neurocognitive disorders and their relationship with functional decline
Source: Front Neurol. 2026 Jul 6;17:1805908. doi: 10.3389/fneur.2026.1805908 (PMC13381444; doi:10.3389/fneur.2026.1805908)
Supplement: Supplementary file 2 [file Data_Sheet_2.PDF]

AD: Predicted FAQ by NPS symptoms over time, among those with at least 4 followup visits

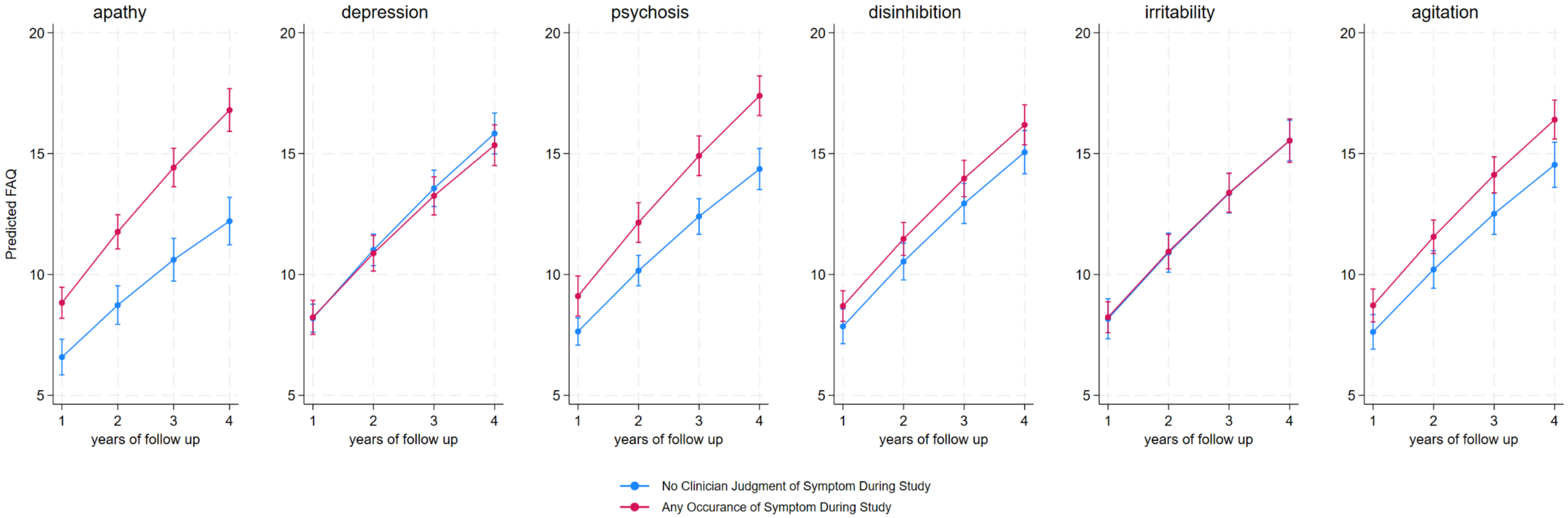

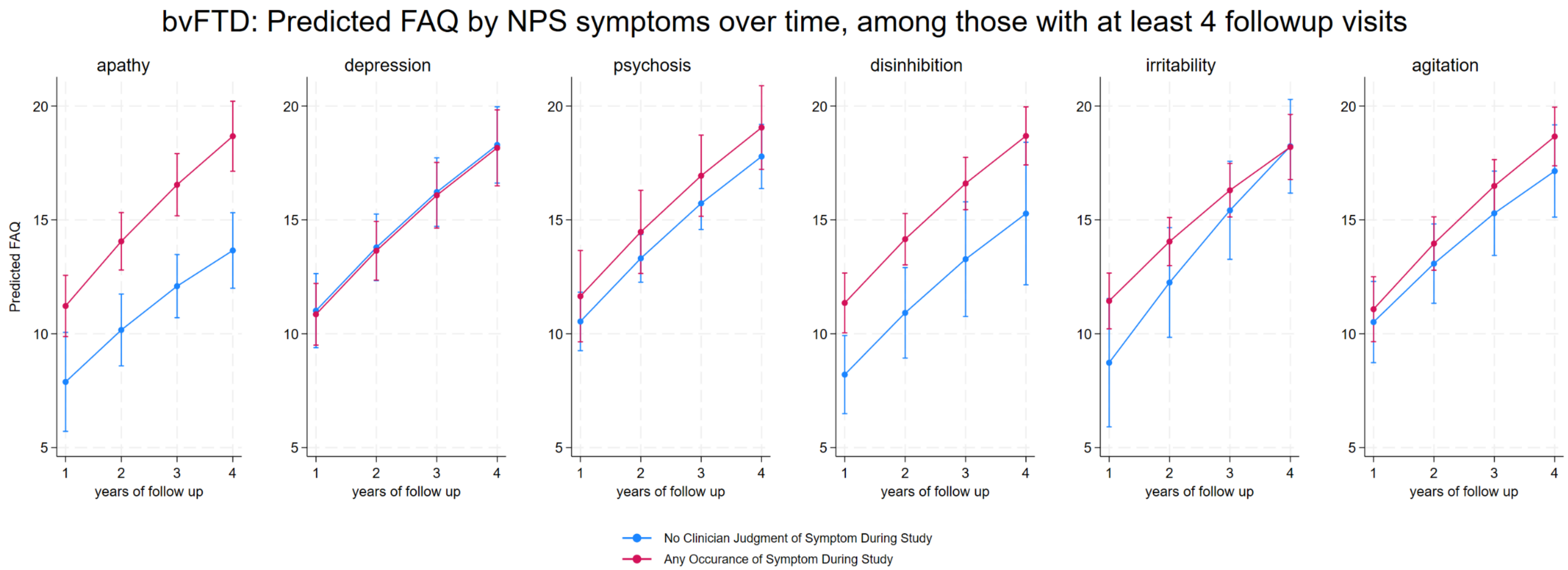
bvFTD: Predicted FAQ by NPS symptoms over time, among those with at least 4 followup visits
apathy
depression
psychosis
disinhibition
irritability
agitation
Predicted FAQ
5
10
15
20
1
2
3
4
years of follow up
No Clinician Judgment of Symptom During Study
Any Occurance of Symptom During Study

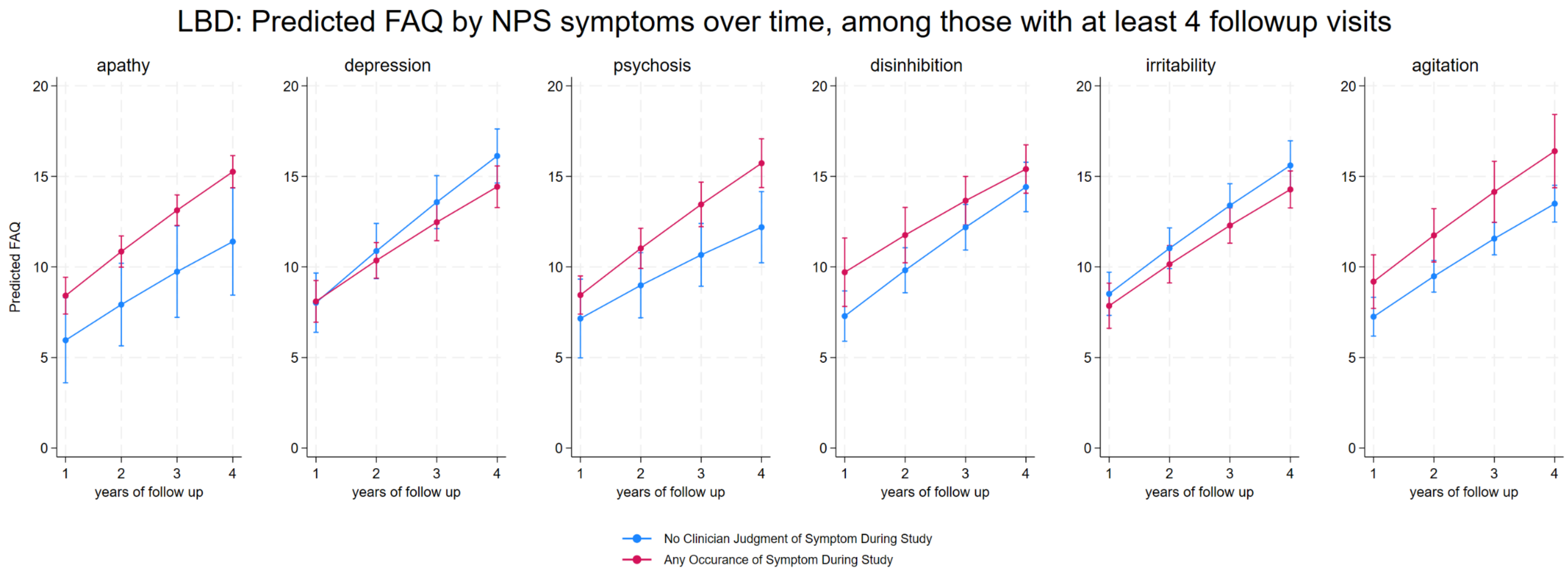
LBD: Predicted FAQ by NPS symptoms over time, among those with at least 4 followup visits
apathy
depression
psychosis
disinhibition
irritability
agitation
Predicted FAQ
years of follow up
No Clinician Judgment of Symptom During Study
Any Occurance of Symptom During Study
