# Supplementary material for: Neuropsychiatric symptom profile in neurocognitive disorders and their relationship with functional decline
Source: Front Neurol. 2026 Jul 6;17:1805908. doi: 10.3389/fneur.2026.1805908 (PMC13381444; doi:10.3389/fneur.2026.1805908)
Supplement: Supplementary file 3 [file Table_1.DOCX]

**Supplemental Table 1. Multivariable results from Linear mixed models (LMM) of the independent relationship between each NPS and functional decline over time in AD, bvFTD, and LBD, among those with at least 3 follow-up visits.**

|  | **AD** |  |  | **bvFTD** |  |  | **LBD** |  |  |
| --- | --- | --- | --- | --- | --- | --- | --- | --- | --- |
|  | **Coefficient** | **SE** | **P>\|z\|** | **Coefficient** | **SE** | **P>\|z\|** | **Coefficient** | **SE** | **P>\|z\|** |
| **Years of follow-up (Time)** | 2.790 | (0.145) | ≤0.001 | 3.865 | (0.753) | ≤0.001 | 2.732 | (0.349) | ≤0.001 |
| **Years of follow-up squared** | -0.156 | (0.009) | ≤0.001 | -0.195 | (0.026) | ≤0.001 | -0.109 | (0.032) | 0.001 |
| **Baseline Dementia Severity (ref: CDR=0.5)** |  |  |  |  |  |  |  |  |  |
| **CDR=1** | 9.909 | (0.484) | ≤0.001 | 6.628 | (0.839) | ≤0.001 | 7.982 | (1.925) | ≤0.001 |
| **CDR=2** | 17.549 | (0.373) | ≤0.001 | 15.392 | (1.255) | ≤0.001 | 17.996 | (1.751) | ≤0.001 |
| **Interaction between baseline CDR * Time** |  |  |  |  |  |  |  |  |  |
| **CDR=1** | -0.547 | (0.091) | ≤0.001 | -0.270 | (0.165) | 0.102 | -0.442 | (0.364) | 0.224 |
| **CDR=2** | -1.711 | (0.107) | ≤0.001 | -1.371 | (0.360) | ≤0.001 | -2.067 | (0.334) | ≤0.001 |
| **Effects on Baseline FAQ from Clinician**  **judged NPS vs. never having the symptom** |  |  |  |  |  |  |  |  |  |
| **Apathy** | 1.365 | (0.260) | ≤0.001 | 3.080 | (1.348) | 0.022 | 1.848 | (0.977) | 0.058 |
| **Depression** | 0.212 | (0.258) | 0.412 | -0.371 | (0.544) | 0.495 | 1.622 | (0.716) | 0.023 |
| **Psychosis** | 0.992 | (0.316) | 0.002 | 0.863 | (1.093) | 0.430 | 1.410 | (1.196) | 0.238 |
| **Disinhibition** | 0.772 | (0.293) | 0.008 | 2.922 | (0.803) | ≤0.001 | 2.689 | (1.189) | 0.024 |
| **Irritability** | -0.083 | (0.369) | 0.822 | 2.725 | (1.468) | 0.063 | -0.054 | (0.772) | 0.944 |
| **Agitation** | 0.948 | (0.332) | 0.004 | 0.981 | (0.906) | 0.279 | 0.058 | (0.926) | 0.950 |
| **Effects on Rate of Change in FAQ from Clinician**  **judged NPS vs. never having the symptom** |  |  |  |  |  |  |  |  |  |
| **Apathy** | 0.790 | (0.096) | ≤0.001 | 0.572 | (0.296) | 0.054 | 0.458 | (0.274) | 0.094 |
| **Depression** | -0.167 | (0.408) | 0.409 | -0.016 | (0.193) | 0.935 | -0.491 | (0.327) | 0.083 |
| **Psychosis** | 0.516 | (0.092) | ≤0.001 | 0.054 | (0.312) | 0.861 | 0.818 | (0.339) | 0.016 |
| **Disinhibition** | 0.098 | (0.067) | 0.143 | -0.061 | (0.321) | 0.850 | -0.338 | (0.334) | 0.311 |
| **Irritability** | -0.031 | (0.080) | 0.696 | -0.920 | (0.414) | 0.026 | -0.319 | (0.196) | 0.103 |
| **Agitation** | 0.250 | (0.080) | 0.002 | 0.291 | (0.199) | 0.144 | 0.450 | (0.344) | 0.190 |
| **Control Variables** |  |  |  |  |  |  |  |  |  |
| **Baseline age** | 0.059 | (0.018) | 0.001 | 0.014 | (0.042) | 0.737 | 0.051 | (0.044) | 0.253 |
| **Male** | -1.780 | (0.218) | ≤0.001 | -0.530 | (0.586) | 0.365 | -0.336 | (0.854) | 0.694 |
| **Race/Ethnicity (reference=Non-Hispanic White)** | 1.583 | (0.330) | ≤0.001 | 0.419 | (1.105) | 0.704 | -0.563 | (1.023) | 0.582 |
| **Years of education** | -0.003 | (0.024) | 0.914 | -0.069 | (0.117) | 0.555 | 0.086 | (0.095) | 0.366 |
| **NACCUDS version** | -0.540 | (0.236) | 0.022 | -0.455 | (0.459) | 0.322 | -0.731 | (0.897) | 0.416 |
| **Years of follow up** | -0.895 | (0.080) | ≤0.001 | -0.876 | (0.165) | ≤0.001 | -1.187 | (0.153) | ≤0.001 |
| **Lives alone at baseline** | -1.339 | (0.277) | ≤0.001 | -0.796 | (1.208) | 0.510 | -3.145 | (1.169) | 0.007 |
| **Referred by professionals** | 1.084 | (0.279) | ≤0.001 | 0.234 | (0.781) | 0.765 | 0.342 | (0.695) | 0.623 |
| **Diabetes** | -0.515 | (0.174) | 0.003 | -0.335 | (0.807) | 0.678 | -1.096 | (0.837) | 0.190 |
| **Hypertension** | 0.017 | (0.186) | 0.926 | -0.646 | (0.724) | 0.372 | -0.289 | (0.823) | 0.726 |
| **Number of medications** | -0.010 | (0.023) | 0.649 | -0.211 | (0.078) | 0.007 | 0.042 | (0.038) | 0.271 |
| **Apolipoprotein ε4 allele (ApoE ε4) (reference=No ε4)** |  |  |  |  |  |  |  |  |  |
| **One ε4** | 0.890 | (0.172) | ≤0.001 | -0.582 | (0.398) | 0.144 | 0.600 | (0.581) | 0.302 |
| **Two ε4s** | 1.683 | (0.299) | ≤0.001 | 4.417 | (1.692) | 0.009 | 2.240 | (1.096) | 0.041 |
| **ApoE missing** | 1.033 | (0.346) | 0.003 | 1.025 | (1.682) | 0.542 | 1.330 | (1.102) | 0.227 |

**Supplemental Table 2. Multivariable results from Linear mixed models (LMM) of the independent relationship between each NPS and functional decline over time in AD, bvFTD, and LBD, among those with at least 4 follow-up visits.**

|  | **AD** |  |  | **bvFTD** |  |  | **LBD** |  |  |
| --- | --- | --- | --- | --- | --- | --- | --- | --- | --- |
|  | **Coefficient** | **SE** | **P>\|z\|** | **Coefficient** | **SE** | **P>\|z\|** | **Coefficient** | **SE** | **P>\|z\|** |
| **Years of follow-up (Time)** | 2.548 | (0.145) | ≤0.001 | 3.392 | (0.796) | ≤0.001 | 2.481 | (0.454) | ≤0.001 |
| **Years of follow-up squared** | -0.139 | (0.010) | ≤0.001 | -0.177 | (0.025) | ≤0.001 | -0.076 | (0.034) | 0.025 |
| **Baseline Dementia Severity (ref: CDR=0.5)** |  |  |  |  |  |  |  |  |  |
| **CDR=1** | 10.094 | (0.506) | ≤0.001 | 6.944 | (0.704) | ≤0.001 | 8.791 | (2.335) | ≤0.001 |
| **CDR=2** | 17.928 | (0.424) | ≤0.001 | 14.621 | (1.312) | ≤0.001 | 19.583 | (1.395) | ≤0.001 |
| **Interaction between baseline CDR * Time** |  |  |  |  |  |  |  |  |  |
| **CDR=1** | -0.550 | (0.091) | ≤0.001 | -0.230 | (0.148) | 0.120 | -0.425 | (0.461) | 0.356 |
| **CDR=2** | -1.669 | (0.103) | ≤0.001 | -1.048 | (0.335) | 0.002 | -2.004 | (0.404) | ≤0.001 |
| **Effects on Baseline FAQ from Clinician**  **judged NPS vs. never having the symptom** |  |  |  |  |  |  |  |  |  |
| **Apathy** | 1.466 | (0.321) | ≤0.001 | 2.769 | (1.328) | 0.037 | 1.995 | (1.465) | 0.173 |
| **Depression** | 0.211 | (0.299) | 0.481 | -0.170 | (0.753) | 0.821 | 0.662 | (1.262) | 0.600 |
| **Psychosis** | 0.942 | (0.395) | 0.017 | 1.056 | (1.194) | 0.376 | 0.544 | (1.409) | 0.700 |
| **Disinhibition** | 0.743 | (0.355) | 0.036 | 3.060 | (0.739) | ≤0.001 | 2.896 | (1.625) | 0.075 |
| **Irritability** | 0.089 | (0.430) | 0.835 | 3.628 | (1.671) | 0.030 | -0.438 | (0.996) | 0.660 |
| **Agitation** | 0.839 | (0.396) | 0.034 | 0.248 | (1.102) | 0.822 | 1.619 | (1.003) | 0.107 |
| **Effects on Rate of Change in FAQ from Clinician**  **judged NPS vs. never having the symptom** |  |  |  |  |  |  |  |  |  |
| **Apathy** | 0.782 | (0.097) | ≤0.001 | 0.562 | (0.341) | 0.099 | 0.465 | (0.406) | 0.252 |
| **Depression** | -0.174 | (0.056) | 0.002 | 0.010 | (0.210) | 0.960 | -0.590 | (0.304) | 0.052 |
| **Psychosis** | 0.522 | (0.095) | ≤0.001 | 0.053 | (0.312) | 0.865 | 0.747 | (0.361) | 0.038 |
| **Disinhibition** | 0.097 | (0.070) | 0.166 | 0.088 | (0.331) | 0.792 | -0.476 | (0.368) | 0.195 |
| **Irritability** | -0.024 | (0.083) | 0.776 | -0.915 | (0.399) | 0.022 | -0.223 | (0.236) | 0.345 |
| **Agitation** | 0.257 | (0.084) | 0.002 | 0.317 | (0.218) | 0.146 | 0.320 | (0.366) | 0.381 |
| **Control Variables** |  |  |  |  |  |  |  |  |  |
| **Baseline age** | 0.067 | (0.019) | 0.001 | 0.055 | (0.059) | 0.347 | 0.087 | (0.058) | 0.136 |
| **Male** | -2.158 | (0.269) | ≤0.001 | -1.222 | (0.902) | 0.175 | 0.813 | (1.198) | 0.498 |
| **Race/Ethnicity (reference=Non-Hispanic White)** | 1.539 | (0.366) | ≤0.001 | 0.592 | (1.834) | 0.747 | -1.503 | (1.060) | 0.156 |
| **Years of education** | 0.003 | (0.029) | 0.929 | 0.096 | (0.129) | 0.458 | 0.174 | (0.123) | 0.158 |
| **NACCUDS version** | -0.462 | (0.239) | 0.053 | -0.241 | (0.491) | 0.623 | -0.432 | (1.135) | 0.704 |
| **Years of follow up** | -0.820 | (0.085) | ≤0.001 | -0.822 | (0.153) | ≤0.001 | -0.703 | (0.230) | 0.002 |
| **Lives alone at baseline** | -1.484 | (0.318) | ≤0.001 | -0.601 | (1.300) | 0.644 | -2.803 | (1.501) | 0.062 |
| **Referred by professionals** | 1.004 | (0.360) | 0.005 | 0.518 | (0.986) | 0.599 | 0.093 | (1.119) | 0.934 |
| **Diabetes** | -0.476 | (0.221) | 0.032 | -1.547 | (1.011) | 0.126 | 0.228 | (0.964) | 0.813 |
| **Hypertension** | -0.141 | (0.199) | 0.478 | -0.937 | (0.850) | 0.270 | -0.728 | (1.218) | 0.550 |
| **Number of medications** | -0.010 | (0.024) | 0.671 | -0.205 | (0.092) | 0.026 | 0.052 | (0.047) | 0.269 |
| **Apolipoprotein ε4 allele (ApoE ε4) (reference=No ε4)** |  |  |  |  |  |  |  |  |  |
| **One ε4** | 1.138 | (0.191) | ≤0.001 | -0.188 | (0.688) | 0.785 | 1.652 | (0.914) | 0.071 |
| **Two ε4s** | 1.916 | (0.281) | ≤0.001 | 4.622 | (1.976) | 0.019 | 5.168 | (1.283) | ≤0.001 |
| **ApoE missing** | 1.222 | (0.443) | 0.006 | 3.430 | (1.648) | 0.037 | 3.303 | (1.621) | 0.042 |
